# Factors Influencing Information Distortion in Electronic Nursing Records: Qualitative Study

**DOI:** 10.2196/66959

**Published:** 2025-04-09

**Authors:** Jianan Wang, Yihong Xu, Zhichao Yang, Jie Zhang, Xiaoxiao Zhang, Wen Li, Yushu Sun, Hongying Pan

**Affiliations:** 1 Department of Nursing Sir Run Run Shaw Hospital Zhejiang University School of Medicine Hangzhou China

**Keywords:** information distortion, electronic health record, qualitative research, ethics, nursing

## Abstract

**Background:**

Information distortion in nursing records poses significant risks to patient safety and impedes the enhancement of care quality. The introduction of information technologies, such as decision support systems and predictive models, expands the possibilities for using health data but also complicates the landscape of information distortion. Only by identifying influencing factors about information distortion can care quality and patient safety be ensured.

**Objective:**

This study aims to explore the factors influencing information distortion in electronic nursing records (ENRs) within the context of China’s health care system and provide appropriate recommendations to address these distortions.

**Methods:**

This qualitative study used semistructured interviews conducted with 14 nurses from a Class-A tertiary hospital. Participants were primarily asked about their experiences with and observations of information distortion in clinical practice, as well as potential influencing factors and corresponding countermeasures. Data were analyzed using inductive content analysis, which involved initial preparation, line-by-line coding, the creation of categories, and abstraction.

**Results:**

The analysis identified 4 categories and 10 subcategories: (1) nurse-related factors—skills, awareness, and work habits; (2) patient-related factors—willingness and ability; (3) operational factors—work characteristics and system deficiencies; and (4) organizational factors—management system, organizational climate, and team collaboration.

**Conclusions:**

Although some factors influencing information distortion in ENRs are similar to those observed in paper-based records, others are unique to the digital age. As health care continues to embrace digitalization, it is crucial to develop and implement strategies to mitigate information distortion. Regular training and education programs, robust systems and mechanisms, and optimized human resources and organizational practices are strongly recommended.

## Introduction

The use of digital technologies in health care is improving the quality of human life, making medical services easier, safer, and more accessible [1]. However, this sociotechnical transformation has also introduced more complex and diverse ethical concerns [2]. Examples of information ethics issues include information disclosure, distortion, alienation, and injustice [3]. Among these, information distortion undermines data accuracy, posing immediate health risks and directly threatening patient safety, which demands significant attention [3]. Although the definition of information distortion varies across different research areas, all studies highlight a common theme: the alteration of information—whether through exaggeration, misinterpretation, or other means [4-6]. This makes it a critical data quality issue. There are a few studies that focused on data quality; although they do not use the word “distortion,” they refer to “accuracy” or “correctness,” terms that relate to distortion [7,8]. However, these studies tended to provide only a general overview of information distortion, treating it as a subset of data quality issues without exploring it in depth. Compared with other quality issues like redundancy or inaccessibility, information distortion could have more severe implications. Inaccurate health data can hinder preventive and therapeutic interventions, potentially compromising patient health outcomes [9]. Research has shown that every 1% reduction in data completeness is associated with a 1.21% increase in missing events [10], the management of which may impose additional burdens in terms of manpower, materials, and finances [11,12]. As health care professionals increasingly rely on electronic medical records (EMRs) to support patient care, the consequences of information distortion become even more pronounced in the digital era. Technology-driven tools, such as clinical decision support systems and algorithms, depend on accurate EMR data to provide reliable guidance and improve health. However, inaccuracies in data can lead to inappropriate medical decisions being perpetuated across multiple patients [13,14]. Furthermore, predictive models built on flawed data may introduce biases, adversely affecting entire populations on a large scale [9]. High-quality data are also indispensable for policymakers, who rely on EMR data for informed decision-making and strategic planning [15].

Research has evaluated the quality of health data in medical documentation, highlighting issues such as accuracy, completeness, and readability, which often intersect with information distortion [16,17]. With similar functions, nursing documentation is also a crucial component of health data quality, serving as a record of a patient’s health data and the care provided [18]. High-quality nursing documentation is essential not only as a tool for nurses to support decision-making and ensure continuity of care but also as a critical reference for doctors to understand the patient’s condition and take appropriate medical actions [19]. Accurate and comprehensive documentation is a legal and ethical obligation, as well as a professional requirement for nurses [20,21]. However, persistent information distortion impedes accurate record-keeping. Information distortion occurs when nursing records fail to accurately reflect nursing practices or the patient’s true condition [3]. Several studies have found that paper-based nursing records often inadequately reflect nursing activities [22,23]. In the current information era, research from Italy found that only 37% of assessments and 45% of interventions are documented in nursing records [24]. Similarly, Korean scholars [25,26] found that certain nursing activities, such as nurse rounding and skin assessments, are often performed but not recorded in electronic nursing records (ENRs). The correlation score between nursing activities and corresponding records was 3.08 on a 4-point scale. Our study (currently under review) further investigated the manifestations of information distortion in ENRs through a qualitative approach (Jianan Wang, BS, unpublished data, January 2025). The findings revealed that information distortion permeates various aspects of ENRs, including assessments, care planning, interventions, incident management, and billing. This is particularly concerning as certain decision-making processes cannot tolerate inaccurate information, which may lead to severe consequences. Given the widespread occurrence of information distortion, understanding the factors influencing this phenomenon is crucial for developing effective interventions to mitigate it. Previous studies have explored contributors to information distortion in paper-based records [22,23], identifying issues such as time constraints and cumbersome charting formats. However, the advent of technology has introduced structural changes to documentation practices, like digital workflows, predefined templates, and integrated decision support tools. These innovations may alter the factors affecting distortion in ENRs due to variations in processes such as data entry, storage, updating, analysis, and auditing. In addition, although factors affecting the quality of both formats of nursing records—such as heavy workloads, nurse fatigue, and hardware shortages—have been studied [27,28], information distortion phenomena manifest in specific and concerted ways within clinical contexts. These phenomena are not merely errors nor omissions; they may arise from complex psychological, organizational, or systemic factors that are difficult to fully uncover in broader quality studies. As no previous research has independently examined the factors associated with information distortion in ENRs, the aim of this study was to investigate the factors contributing to information distortion phenomena in ENRs in China. Our results will help identify potential risks associated with ENRs and contribute to the development of targeted preventive strategies to reduce information distortion.

## Methods

### Study Design

We chose a qualitative descriptive approach for this study, as it is well-suited for obtaining direct insights from participants about a poorly understood phenomenon [29,30]. Semistructured interviews were conducted to facilitate in-depth discussions of participants’ perspectives. The study adhered to the Consolidated Criteria for Reporting Qualitative Research guidelines [31] (Multimedia Appendix 1).

### Setting

This study was conducted in a Class-A tertiary hospital in Zhejiang Province, China. The hospital has been using electronic documentation for over 20 years and integrated a nursing decision support system (NDSS) into its nursing information system in 2016. The hospital’s information system was independently developed in-house, tailored specifically to meet the institution’s unique operational needs and workflows, ensuring full control over system customization, integration, and ongoing updates. The hospital’s advanced level of digitalization has provided nurses with a comprehensive technological environment, giving them extensive experience with the system, which is invaluable for gaining insights into issues related to information distortion.

### Ethics Approval

Approval was obtained from the Ethics Committee of Sir Run Run Shaw Hospital, Zhejiang University School of Medicine (approval number: 2024 Research No. 0270). Written informed consent was obtained from all participants by one of the authors (JW) prior to data collection. No participants opted out of the study. All data transcripts for analysis were deidentified.

### Participants and Recruitment

Both purposive and snowball sampling methods were used. Purposive sampling is a common technique in qualitative descriptive studies, selecting individuals who have relevant experience with the phenomenon of interest [32]. To ensure interviewees provide valuable insights into the nuances of information distortion, we decided to select members of the Information Committee as participants. Each department has a representative in the committee. The committee members are nurses actively involved in clinical practice who also take on additional responsibilities, such as addressing nurses’ concerns about system usage and sharing technical knowledge. Given their roles, these nurses are more likely to have encountered challenges and ethical dilemmas related to electronic documentation. Their insights are invaluable for uncovering the underlying causes of information distortion, which may not be as evident to nurses with less experience in this area. Considering that the information systems vary across different specialties, we conducted maximum variation sampling based on department categories after confirming members’ willingness to participate to ensure that the selected participants encompassed as many aspects and dimensions of clinical nursing work as possible. Participants were not only asked about their own experiences but also about the experiences of their colleagues they had observed. This approach allowed for the inclusion and comparison of diverse perspectives, making the insights into factors influencing distortion in ENRs more representative of the broader nursing community. Snowball sampling involves participants recommending additional participants from their acquaintances, thereby continuously introducing new potential participants. We invited Information Committee members to introduce nurses who had been involved in clinical informatics–related work or projects as interviewees. Targeted nurses were invited through the hospital’s internal network. Inclusion criteria for nurses were (1) registered nurses employed at the hospital for at least one year and (2) voluntary participation with signed informed consent. Nurses who were absent during the study period (eg, on leave, vacation, or away for training) were excluded. Nurses from other hospitals who were present for training were also excluded, but participant nurses were encouraged to share relevant experience from their previous employment at other hospitals. Recruitment continued until data saturation was achieved, meaning no new substantive themes emerged from additional data collection [33].

### Data Collection

Face-to-face interviews were conducted in the coffee shop or the demonstration classroom in the hospital between January 9, 2024, and April 20, 2024, at times convenient for the participants. The interviews were conducted by one author (JW), a female PhD student trained in qualitative research methods. Only the participants and the interviewer were present during the interviews. The interviewer had no prior relationship with the participants, which helped minimize biases and address potential ethical concerns. Before the interviews began, the interviewer explained the concept of information distortion and the purpose of the study to ensure that participants had the necessary background to provide informed and accurate responses. Informed consent materials were provided, and written consent was obtained from all participants.

As part of the interview process, participants completed a short questionnaire about their personal characteristics, including gender, age, education, post, professional title, work experience, and work department. A topic guide with open-ended questions (see Multimedia Appendix 2) was used to ensure comprehensive coverage of relevant topics during the interviews. The interview questions were developed by reviewing the existing literature and absorbing expert opinions. To ensure validity, the guide was pretested by 2 nurses and revised based on their feedback. Participants were initially asked to describe instances of information distortion they had encountered or observed in clinical settings. Subsequently, they were prompted to identify factors contributing to these phenomena. Follow-up questions were tailored to participants’ responses to encourage deeper elaboration. The next section focused on coping strategies to mitigate information distortion. During this discussion, participants often provided additional insights into influencing factors. Finally, participants were invited to share any additional thoughts or address overlooked aspects before concluding the interview. The interviewer took field notes during the interviews to supplement the data and highlight key moments [34]. By the 14th interview, no new insights emerged, and data began to repeat, indicating that further data collection was unnecessary. Thus, data saturation was achieved after 14 interviews. Interviews lasted between 35 minutes and 97 minutes. All interviews were audio-recorded with participants’ permission, transcribed verbatim, and checked by participants. No repeat interviews were conducted.

### Data Analysis

Inductive qualitative content analysis was used for data analysis, encompassing 3 phases: preparation, organizing, and reporting [35], in which codes and categories were directly drawn from the data [36]. The recorded interviews were transcribed by one author (JW) then verified by the participants. The preparation phase started with selecting the unit of analysis [35]. Since the aim of this study was to explore influencing factors of information distortion, we especially considered participants’ words or a set of sentences and paragraphs addressing influencing factors as meaning units. Two researchers (JW and YX) read all relevant data repeatedly to achieve immersion and gain an overall understanding of the content. The organization phase included open coding, creating categories, and abstraction [35]. The same two researchers started open coding by independently reading transcripts line by line, extracting texts with similar meanings, and grouping them into fewer content-related codes to identify influencing factors [37]. The researchers also made notes of their initial impressions and thoughts regarding the codes. The codes were collected to the coding sheets. The two then synthesized their coding sheets and independently sorted them into more formalized subcategories, using quotations derived from the interviews. Next, they compared each subcategory, merging those with the same meanings and discussing those with differences. Discrepancies that could not be resolved through discussion were addressed within the research group until consensus was reached. Rearranging subcategories also occurred in this process. Finally, based on the relationships between subcategories, researchers collaboratively grouped a larger number of subcategories into a smaller number of categories. All data management and analyses were conducted in NVivo 12 (QSR International). The interviews were conducted in Mandarin, and only the directly quoted passages were translated into English for inclusion in the paper. The translation was performed by a professional translator fluent in both languages. The translation was reviewed by the research team to ensure the meaning of the original Mandarin quotes was faithfully preserved in the English translation.

### Rigor

The rigor of this study was ensured by credibility, transferability, dependability, and confirmability [38]. Credibility was ensured by having two researchers independently code the data, bracketing personal biases. Furthermore, member checks were conducted by inviting participants to review and provide feedback on generated categories and subcategories. Transferability was ensured by providing a detailed description of the participants’ inclusion and exclusion criteria, data collection and analysis procedures, and participant characteristics, enabling readers to relate findings to their own contexts. Dependability was ensured through the detailed methodological documentation, where all headings, subcategories, and categories were documented at each step, allowing others to assess the process. Confirmability was ensured by maintaining a journal to track researchers’ evolving thoughts, biases, and reflections. The final categories and subcategories were reviewed by the entire research team to ensure coherence, which also increases the objectivity and confirms the accuracy of the findings.

## Results

### Participants

The demographic characteristics of the participants are presented in Table 1. We interviewed a total of 14 nurses, including clinical nurses, advanced practice nurses, and nurse educators. In terms of professional title, 3 participants were primary nurses, while 11 were charge nurses. All participants provided direct patient care, with charge nurses also responsible for overseeing junior nurses and mentoring interns, trainees, and new colleagues. Their working departments varied and included the intensive care unit, surgery, and internal medicine. All interviewees were female and aged 26 years to 37 years, with work experience ranging from 4 years to 14 years. Most of them (12/14, 86%) had a bachelor’s degree, and 2 of them had a master’s degree.

**Table 1:** Characteristics of participants (N=14).

Characteristic								Values, n (%)
**Gender**								
	Female						14 (100)	
**Age (years)**								
		25-29					3 (21)	
		30-34					7 (50)	
		35-39					4 (29)	
**Education**								
				Bachelor’s degree			12 (86)	
				Master’s degree			2 (14)	
**Post**								
			Clinical nurse					8 (57)
			Advanced practice nurse					3 (21)
			Nurse educator					3 (21)
**Professional title**								
				Primary nurse			3 (21)	
				Charge nurse			11 (79)	
**Work experience (years)**								
						1-5		2 (14)
						6-10		8 (57)
						11-15		4 (29)
**Work department**								
						Intensive care unit		1 (7)
						General surgery		3 (21)
					Medical oncology		1 (7)	
					Respiratory medicine		1 (7)	
					General internal medicine		1 (7)	
					Endocrinology		1 (7)	
					General ward		1 (7)	
					Cardiac surgery		1 (7)	
					Rheumatology and immunology		1 (7)	
					Neurosurgery		1 (7)	
					Neurology		2 (14)	

### Factors Influencing Information Distortion Behaviors

**Figure 1:**
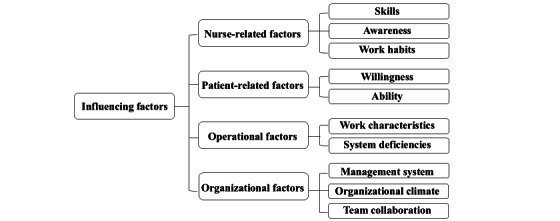
Categories of influencing factors.

We generated 4 categories and 10 subcategories about factors influencing information distortion phenomena in ENRs through the interviews (see Figure 1). Illustrative quotes from participants are provided in Multimedia Appendix 3.

#### Nurse-Related Factors: Skills

The lack of certain skills may contribute to nurses’ information distortion phenomena. With the advancement of technology, such as NDSS and the copy-and-paste function, nurses may rely too heavily on system-generated advice or records from colleagues without critical evaluation, increasing the likelihood of distorted records. Additionally, a lack of multitasking and time management skills, particularly among junior nurses, can make them “busier and slower at handling issues,” leading to omissions or errors in their ENRs that fail to accurately reflect patients’ conditions. Nurses also indicated that job familiarity influences these phenomena. New nurses, who are unfamiliar with their duties, are more prone to making mistakes in their records, while experienced nurses, who may be overconfident in their abilities, might neglect to recheck their entries, resulting in inaccuracies. Furthermore, the introduction of new system designs can also lead to errors in records due to unfamiliarity.

#### Nurse-Related Factors: Awareness

Work attitude plays a significant role in influencing information distortion phenomena. Nurses who are meticulous in their work tend to produce more accurate records, while those who are more casual and indifferent are more likely to write distorted records. The perception of information importance also affects the accuracy of their ENRs. Nurses reported that they pay more attention to critical aspects related to patient safety, frequently audited items, and department-specific issues. These areas are documented more carefully, resulting in fewer inconsistencies. However, minor issues not directly related to a patient’s illness, such as a small skin condition, may go unrecorded even if noticed. Additionally, perceived threats, such as fear of medical disputes or judgment from others, can influence the occurrence of distortion phenomena. In terms of medical disputes, nurses indicated two possible reactions: They might document all nursing practices in ENRs to verify their workload and reduce disputes, which is a great phenomenon, or they might falsify records to appear perfect, given that ENRs serve as the primary evidence of their work. Concerns about others’ judgment, particularly regarding incident reporting, also play a role. Nurses may worry about negative repercussions, thinking, “Will the head nurse blame me?” or “How could you make such a basic mistake?” As a result, they might cover up mistakes to protect their image. Furthermore, due to easily accessible entries in their colleagues’ records given by digital technologies, nurses may give in to professionalism or authority, leading to potential information distortion. Nurses might feel less confident recording their true observations if more experienced colleagues have documented differently, or they might question their own judgments when comparing their records to those of others so that they might hide their real thoughts.

#### Nurse-Related Factors: Work Habits

Specific work habits can contribute to information distortion. Many nurses use the copy-and-paste function to enhance work efficiency. Some nurses prefer to copy records from the previous shifts then adjust them after completing their nursing activities. However, this habit can lead to forgetting to make the necessary adjustments, resulting in identical records across shifts. Additionally, when nurses do not take the time to review their records, errors may go unnoticed. Nurses also mentioned that fixed work habits can pose a problem. When unexpected events occur, they might be omitted from the records simply because they fall outside of the usual routine.

#### Patient-Related Factors: Willingness

Patients are a primary source of information, and much of the content in ENRs is based on their complaints. In clinical practice, patient-reported information is typically reflected accurately in ENRs. However, when patients are uncooperative, such as by intentionally withholding information or being unwilling to share, nurses may face challenges in accurately assessing their condition.

#### Patient-Related Factors: Ability

Even when patients are highly willing to cooperate, their lack of certain objective abilities can still hinder the accuracy of information. Nurses mentioned difficulties with patients who speak dialects or foreign languages, where communication often becomes challenging and prone to misunderstandings, leading to more frequent instances of distorted records. In addition, cognitive and comprehension abilities can also affect the accuracy of ENRs. Cognitive decline is common among older adult patients and hinders effective communication. Under this circumstance, if family members are not well-informed about the patient’s condition, nurses may face challenges with obtaining accurate information, potentially resulting in distorted records.

#### Operational Factors: Work Characteristics

Nurses face a well-documented heavy workload. The implementation of information systems and electronic records is designed to streamline clinical workflows and enhance efficiency [39]. However, interviews revealed that nurses continue to feel burdened by both documentation and clinical duties. As a result, they often complete their documentation hastily, leading to potential omissions and inaccuracies. Due to their heavy workload, nurses also admitted to using shortcuts in documentation, further compromising the accuracy of their records. Certain characteristics of nursing records also contribute to information distortion. For instance, forms assessed frequently, such as critical care forms updated hourly, tend to be more accurate than those reviewed only once daily, as frequent access facilitates the identification and correction of mistakes. Moreover, assessments with subjective criteria are more prone to discrepancies, as individual nurses may have differing interpretations, leading to a lack of consensus on the patient’s condition. Frequent interruptions also exacerbate information distortion, as nurses must address these issues before returning to their documentation, potentially resulting in inaccuracies in both timing and content.

#### Operational Factors: System Deficiencies

Nurses reported that inadequate information system design hinders accurate documentation. Limited system options and insufficient space for remarks often prevent them from fully capturing their observations. Even with remark sections, redundant entries increase the risk of distortion. Additionally, poor integration between the clinical and nursing information system and between systems in the emergency room and wards hampers the proper display and submission of information, making it difficult for ENRs to reflect the actual situation accurately.

#### Organizational Factors: Management System

Nursing leadership strongly influences staff behaviors [40]. Nurses noted that their leaders’ focus impacts how carefully they document. When leaders stress the importance of accurate ENRs, nurses become more meticulous, reducing distortion. Direct leaders, who “focus on specific areas,” were seen as more influential than higher-level leaders. Hospital regulations also impact documentation. Nurses reported that mismatch between regulatory requirements and clinical realities often lead them to adjust records. Areas without strict rules or audits receive less attention, as inconsistencies are seen as acceptable. In addition, the audit system has a dual impact on information distortion. The National Healthcare Security Administration enforces regulations to ensure the proper use of medical insurance funds and conducts irregularly electronic audits of records. Through digital technologies, these audits are conducted more frequently, discreetly, and cost-effectively. Although this system encourages detailed and accurate documentation to meet compliance standards, it can also lead some nurses to falsify records to secure higher reimbursements.

#### Organizational Factors: Organizational Climate

The organizational atmosphere strongly influences nurses’ behaviors, particularly through conformity. The nurses interviewed indicated that, even if they recognized information distortion, they would gradually take it less seriously if the organization did not prioritize the issue. Conversely, in organizations where accuracy is emphasized, nurses collectively focus more on precise ENRs. Additionally, some nurses admitted to distorting records to protect the organization’s reputation, either by avoiding incident reports or shifting blame to other departments to lower the rate of adverse events. Group pressure also plays a role; when confronted with numerous colleagues pointing out issues in their ENRs, they were more likely to yield and alter their records, even if they had originally documented the truth.

#### Organizational Factors: Team Collaboration

Patient care is definitely a product of teamwork across health care professionals [41], and collaboration between multiple professionals could impact information distortion. Nurses reported that their communication with doctors plays a role in affecting the accuracy of ENRs. They indicated that doctors often communicate directly with patients rather than with them, leaving nurses unaware of the patients’ true condition. When nurses ask patients about what the doctor said, it may lead patients to question the nurses’ professionalism, preventing nurses from obtaining accurate information at that moment. Additionally, nurses were often hesitant to address questionable orders from doctors, either due to concerns about overstepping boundaries or for inconvenience. Consequently, nurses might record inaccurate ENRs based on these orders or ignore them entirely, leading to inconsistencies with reality. Furthermore, doctors occasionally review nursing records and point out documentation issues, which can act as a deterrent, prompting nurses to prioritize accuracy in their documentation. Nurses have noted that electronic formats result in these reviews occurring more frequently. Some nurses also acknowledged intentionally underrating patients’ conditions to draw more attention from doctors, thereby enhancing patient safety.

Similar patterns were observed in collaboration with nursing colleagues; for instance, failing to mark a care plan as completed was sometimes used as a strategy to maintain colleagues’ focus. Additionally, interactions with nursing assistants can also affect information distortion. If nursing assistants lack adequate expertise or effective communication with nurses, misunderstandings about the patient’s condition may arise, resulting in inaccurate ENRs.

## Discussion

### Principal Findings

Using a qualitative approach, we identified various factors contributing to information distortion in ENRs. It is acknowledged that information distortion is not limited to ENRs; similar issues have been observed in general EMRs [16,17]. However, our findings reveal specific content in the nursing context. Some distortions existed in nursing records long before the advent of information technologies [22,23], and some influencing factors have remained constant across paper-based and digital records. However, with the introduction of digital technologies, the phenomena have different manifestations, and some influencing factors are unique to the digital era.

First, although technological advancements provide conveniences, such as enhanced and accelerated professionalism decision-making with the use of an NDSS, they can also increase nurses’ laziness, leading nurses to rely more on system-generated advice rather than their own judgment. Noting that data-driven tools cannot be accurate all the time, the overreliance on these tools can increase more distortion. Second, technology also brings changes to nurses’ work habits, such as copy and paste; although it saves time, it can exacerbate record distortion if nurses do not monitor it closely. Third, compared with paper-based records, the writing of ENRs happens through information systems, which means that the design of the information system plays a key role in documentation. A poorly designed system may limit nurses’ options, introduce redundant entries, or obstruct information exchange, which could potentially hinder accurate recording by nurses and worsen information distortion. Fourth, digital records have made entries more accessible; with just a few clicks, users can get targeted information from their own records as well as others. With the increasing interaction or comparison with others’ records, the accuracy of records might be doubted, especially for those who are less confident or give way to authority. However, electronic formats also improve record quality in some ways. This kind of operation promises consistent checks and makes record auditing easier. When used effectively, this can enhance supervision and reduce information distortion.

Therefore, digital technologies can be viewed as a double-edged sword in relation to information distortion. Given that digitalization is inevitable, it is crucial to develop strategies to address these issues. We propose several measures to enhance the quality of ENRs.

First, regular training should focus on critical thinking, multitasking skills, new procedures, and onboarding. This study shows that skill gaps in these areas lead to record distortion, task delays, and poor performance [42,43]. Targeted improvement initiatives are crucial. Moreover, communication training emphasizing empathy and nonverbal skills should be conducted to build trust and improve the accuracy of patient information. Teamwork training across health care professionals is equally important for maintaining accurate records and fostering a collaborative environment. Educational programs should also highlight the consequences of medical disputes and the importance of accurate records. Continuing education should extend to both nurses and nursing assistants to enhance knowledge, confidence, and collaboration.

Second, certain mechanisms should be improved. For example, routine file inspections conducted by administrators, along with penalties for falsifying records, can serve as deterrents against misconduct [44]. Providing patients access to their records (eg, via Open Notes [45]) could also enhance the accuracy of documentation, as patients can help identify errors. Additionally, establishing a communication and feedback system could address usability issues in the nursing information system and resolve any regulatory concerns. Research has shown that a positive work attitude influences the quality of nurses’ documentation [46]. Therefore, implementing recognition programs to highlight exemplary practices is recommended to improve nurses’ attitudes toward recordkeeping and reduce distortion.

Third, given the pivotal role of information technologies in nursing, tools like translation services and wearable devices can help minimize patients’ communication barriers during data collection [38]. Artificial intelligence can improve ENR quality by using machine learning to detect record errors and large language models to analyze clinical text, ensuring data completeness and accuracy [47-49]. Additionally, information systems must be updated to align with clinical requirements. Enhancing interoperability will facilitate cross-professional checks, reducing uncertainties and omissions in ENRs [50]. Informatics nurses should promote collaboration between technicians and clinical staff to enhance the usability of intelligent devices and information systems, mitigating technology-related challenges [51].

Fourth, heavy workload significantly impacts the quality of ENRs. High patient-to-nurse ratios compel nurses to complete tasks, including their documentation, more hastily [52]. Clinical nurses spend 17% to 27% of their time on ENR-related tasks, surpassing the time allocated to direct patient care or communication [53,54]. Therefore, increasing nurse staffing and simplifying documentation processes are essential to reduce their burden [55,56]. This would facilitate more thorough documentation and decrease the likelihood of information distortion. In addition, the use of medical scribes in the United States—unlicensed individuals who enter data into EMRs under clinicians’ supervision—has been growing [57]. These scribes can also assist with ENRs, helping to reduce nurses’ documentation workload and allowing them to focus more on maintaining the accuracy of records.

Finally, management practices are closely associated with organizational procedures, and effective leadership serves as a potential catalyst for fostering positive team behaviors [58,59]. Therefore, it is essential that leaders place greater emphasis on addressing information distortion issues, as this will influence staff attitudes. Leaders should also promote an open and positive organizational climate by establishing core values and behavioral guidelines that emphasize trust, learning, and accountability while reducing feelings of blame or fear [60]. The role of direct leaders, as identified in this study, is essential to the whole team. Therefore, they are responsible for not only conveying information from upper management to the nursing staff but also for leading by example, embodying the values and behaviors expected within the team.

### Limitations

This study has several limitations. First, the sample size may appear small. However, saturation was reached, and this sample size aligns with the typical saturation range of 12 to 20 interviews, as suggested by Guest et al [61]. Second, interviewees were women from one hospital, and only those with specific training on electronic notes or information technology were invited. This may have contributed to bias, as findings from this study may not apply to other settings. However, given the predominance of female nurses in the Chinese workforce, the limitation of gender was inevitable. We used maximum variation to gain perspectives from various departments, which may help increase transferability. In addition, participants from the Information Committee engage in more frequent discussions with other nurses about information systems, so their perspectives represent those commonly mentioned by most nurses in their departments. Furthermore, during the interviews, we also asked about their colleagues’ experiences, allowing for the sharing and comparison of other nurses’ opinions, which helps improve the generalizability of the findings. Third, although we discussed management strategies and leadership styles, we did not include nurse leaders in our interviews, which may have limited our understanding of how leadership affects information distortion. However, the aim of this study was to explore clinical nurses’ perspectives about influencing factors of information distortion in ENRs. Since nurse leaders in China do not participate in direct care, they are primarily concerned with macro-level management and policymaking rather than the detailed aspects of documentation behaviors, which may not fit the objectives of this study. Future research could incorporate the perspectives of male nurses and nurse leaders from more hospitals for a more comprehensive view of influencing factors in information distortion.

### Conclusions

We conducted a qualitative study using semistructured interviews with Chinese nurses to explore the influencing factors of information distortion in ENRs. The analysis identified that these phenomena are driven by nurse-related, patient-related, operational, and organizational factors. To reduce the likelihood of information distortion, further development is needed in training, mechanisms, techniques, human resources, and organizational practices.

## Appendix

Multimedia Appendix 1 Consolidated Criteria for Reporting Qualitative studies (COREQ) checklist.

Multimedia Appendix 2 Topic guide.

Multimedia Appendix 3 Illustrative quotes from participants.

## Abbreviations

EMR: electronic medical record
ENR: electronic nursing record
NDSS: nursing decision support system

## Data Availability

All data generated or analyzed during this study are included in this published article and its supplementary information files.
