# Supplementary material for: Factors Influencing Information Distortion in Electronic Nursing Records: Qualitative Study
Source: J Med Internet Res. 2025 Apr 9;27:e66959. doi: 10.2196/66959 (PMC12018866; doi:10.2196/66959)
Supplement: Multimedia Appendix 2 [file jmir_v27i1e66959_app2.docx]

**Topic guide**

Introduction

⦁Provide an introduction with background of the study, aims and structure of the interview.

⦁Ensure informed consent is obtained and permission for audio recording is granted.

Experiences

⦁Have you ever encountered instances where nursing records did not match reality in your work? Can you share some notable examples?

1. When did that happen?
2. Where were you at that time?
3. How long did it occur?
4. What actions did you take?

⦁Have your colleagues ever experienced this phenomenon?

⦁Has anyone ever noticed this phenomenon?

Influencing factors

⦁Why do you think this phenomenon occurs?

1. Personal factors?
2. Environmental factors?

⦁To better address this issue, what efforts do you think can be made?

1. What can be done in terms of regulation?
2. What can be done in terms of system design?
3. What other measures can be taken?

Closing

⦁Is there anything else about this topic you would like to add?

⦁Thank you statement and closing.
