# Supplementary material for: Factors Influencing Information Distortion in Electronic Nursing Records: Qualitative Study
Source: J Med Internet Res. 2025 Apr 9;27:e66959. doi: 10.2196/66959 (PMC12018866; doi:10.2196/66959)
Supplement: Multimedia Appendix 3 [file jmir_v27i1e66959_app3.docx]

**Illustrative quotes from participants**

| Category | | Sub category | | Participant quotes | |  |
| --- | --- | --- | --- | --- | --- | --- |
| Nurse-  related factors | | Skills | | Assessment records are just copied from others without any changes. (Nurse 10[N10])  Now the decision support system is way better than when it first started, so sometimes I just save and confirm without thinking too much. (N12)  They (junior nurses) are busier and slower at handling issues. When they’re juggling multiple tasks, they might miss things in their assessments. (N7)  Experienced nurses often think they already know it (tips for the assessment items on the NIS) all and don’t bother checking…At first, there wasn’t a section for a nursing care plan, but then one day it was suddenly added, and people often forgot to propose that (a care plan) at that time. (N2) | |  |
|  | | Awareness | | Some colleagues do a great job, but others figure that if they just finish the documentation, it doesn’t matter if it’s done well. (N7)  We focus more on neurological signs since it’s our specialty and we have regular quality checks, so we have fewer inaccuracies…They (junior nurses) might think, “Someone fell on my shift. Will the head nurse blame me?”…“How could you make such a basic mistake?” (N12)  If the patient’s skin is damaged, she (the nurse) might notice that but think it’s not really related to the illness, so she doesn’t note it in the records. (N2)  If someone later checks the records and sees that a patient had a fever and the medication was given an hour late, it could lead to a lot of issues. (N11)  We’ve done this nursing procedure, if you don’t document it, it won’t show your workload or what you’ve actually done for the patient. (N13)  If the previous shift had a more experienced nurse who didn’t write something down, the less experienced nurse on the next shift might think, “They didn’t write it, so maybe it’s not necessary.” This can lead to self-doubt, and they might not write it down either. (N3) | |  |
|  | | Work habits | | Sometimes nurses might copy things over before rounding, thinking they can correct them later. But if they get busy, they might forget. (N12)  New colleagues haven’t developed excellent habits yet. If you go back and look over what you’ve written, you’ll definitely spot any mistakes. (N9)  Our work habit is to charge for services in the morning. So if a nebulizer treatment starts around noon, we won’t charge for that day, just once. (N1) | |  |
| Patient-  related factors | | Willingness | | When patients are admitted, we use the address on their ID card, which might be from 10 years ago. When we ask, they might say they still live there, but in reality, they might have moved somewhere else. (N2)  Last time we had a patient who didn’t answer any questions we asked…You cannot write up a record when you can’t get any information. (N4) | |  |
|  | | | Abilities | | Many family members don’t even know how many implants their loved one has. If the patients can’t explain it clearly and their family doesn’t know, and we can’t tell by looking, it’s really hard to know the truth. (N12)  We’ve had a lot of foreign patients in our department recently, they speak in a way we can’t understand, so our records sometimes are inaccurate. (N2)  If an out-of-town nurse is admitting them (elderly patients who don’t speak Mandarin), it gets hard because she can’t understand. We later realized some records were wrong because she misheard them. (N13) | |
| Operational factors | | | Work characteristics | | There are so many documents to handle that it’s impossible to check each one carefully. (N7)  If we mark a family member (regarding who to educate), we have to explain their relationship to the patient, it is a hassle. So sometimes when the patient cannot communicate, we still mark them, even though we often end up talking to the family member…In surgery, there are a lot of interruptions, like getting saline ready or patients going for surgery. With all these small tasks, it can mess up the records we are supposed to note. (N8)  Assessment forms are filled out once a day, so I might just copy it in the morning, and that’s it…I fill critical care forms out every hour. Each time I open it, I see the previous entries, and I might notice errors. (N12)  We have a young patient with pancreatitis. I saw him marked as frail. But then I saw him walking around, oh he doesn’t seem frail to me. (N1) | |
|  | | | System deficiencies | | The only option available (in the care plan) is “PC (potential complication) Paralytic Ileus.” But that’s just one type of bowel obstruction. I want to address it as a general bowel obstruction…And there is no place to add notes. (N5)  Every time the patient’s condition changes, you need to update all the places, but there are often mismatches between these sections. (N7)  If a doctor’s order is issued after 4 PM, our nursing infusion system can’t print the infusion cards. But we still have to give the infusion, so it’s like doing it without an official order in the system. (N3)  When a patient is transferred to the ER (emergency room), we can’t submit the inter-department transfer form to ER, they use different systems. (N1) | |
| Organizational factors | | | Management system | | Higher-level leaders might oversee the whole hospital, but the direct leader is focused on our specific area and handles issues more closely. If she cares about it, we’ll definitely take it seriously. (N10)  If a rule says it has to be done exactly every 15 minutes, it’s not always practical in a clinical setting. You can’t be perfect and check every 15 minutes on the dot; there will always be a few minutes’ difference. (N11)  No one checks whether your nursing plan is completed. There’s no verification system. So we just complete it without any concern. (N5)  We also sometimes score high-risk pressure ulcers at 18 or below on purpose so we can charge for them, since only scores of 18 or below qualify for high-risk pressure ulcer fees…Because charges are closely tied to everyone’s income. (N1) | |
|  | | | Organizational climate | | Even if you notice the problem (about the information distortion) and ask an experienced colleague about it, they’ll say, “We’ve always done it this way, everyone does it like this.” So you feel like you can’t change it on your own because everyone treats it as a set way of doing things. (N3)  Because in our department, even a tiny issue is treated like a huge deal. So, things like that (distorted records) almost never happen. (N4)  We tend to blame ourselves, feeling like we’ve added a “blemish” to our department. So some nurses might be reluctant to report incidents…When handing over to the ICU (intensive care unit), if the ICU sees any redness on the skin, they’ll have you fill out a report, even if it’s not actually a pressure ulcer. It feels like they’re pushing us to document everything to shift responsibility away from themselves…they (ICU nurses) insist that you fill it out, and with four or five of them surrounding you, all saying it is, those with a softer personality might not dare to disagree. (N6) | |
|  | | | Team collaboration | | The doctor might tell the patient that their condition is serious, but if we don’t hear that, we won’t know…When we ask patients about what the doctor said, they might respond with, “How could you not know this?” (N6)  If the VTE (deep vein thrombosis score) (doctors’ work) is wrong, then our risk level assessment is wrong too. But we usually don’t talk to them (doctors) about it because we feel like it’s more the doctor’s area and not really our place to correct. (N8)  Our doctors often mess up when writing orders and have to redo them…Communicating with them is such a pain…So during the time when there’s no order, we still use restraints and keep asking the doctor to write the order. (N14)  He (the doctor) thinks we’re not recording intake and output correctly because many shock patients sweat a lot, and we often forget to record the amount of sweat. Now that we know, we will pay attention to that and it won’t happen again. (N13)  To use restraints, we need a doctor’s order. Before the doctor gives the order, the patient’s risk score has to be pretty high. So we might rate them higher than actual to make sure they get the restraints. (N12)  Once I mark a plan as completed, the next nurse might not pay attention to it anymore. So I never mark it (as completed). (N3)  If they (nursing assistants) find that a tube is missing, they won’t tell you. I might ask them, “Is there nothing in this tube?” and they’ll say, “If I didn’t record anything, that’s empty.” So I might think the tube is just empty, but it actually exists. (N2) | |
